# The Treatment of Caries of the Spine

**Published:** 1897-11

**Authors:** A. H. Tubby

**Affiliations:** M S., Lond, F. R. C. S., Eng.; Assistant Surgeon and in charge of Orthopedic Department, Westminster Hospital; Surgeon to Out-patients Evelina Hospital for Sick Children; Surgeon to the National Orthopedic Hospital, etc.


### THE TREATMENT OF CARIES OF THE SPINE.

By A. H. TUBBY, M S., LOND, F. R. C. S., ENG.

**Assistant Surgeon and in charge of Orthopedic Department, Westminster Hospital; Surgeon to Out-patients Evelina Hospital for Sick Children; Surgeon to the National Orthopedic Hospital, etc.**

In spite of the severe nature of the spinal disease, much good may be done, and cure often effected. If the case comes under treatment in its early stages, deformity may be prevented or reduced to a minimum. In the absence of complications, cure will most readily be obtained in the cervical, lower dorsal and lumbar regions; less readily and with more difficulty in the upper and mid-dorsal regions. The method of cure is by ankylosis, and when the disease is treated early and thoroughly the number of vertebræ ankylosed is small, the patient having a very useful back. I know of one who is able to climb difficult mountains, despite ankylosis of the four upper lumbar vertebræ. In his case there is no deformity. The treatment may extend over years, but the ultimate results are often such as to give solid satisfaction to the surgeon. Treatment may be described as general and local.

*General Treatment.*—Fresh air and sunshine are most valuable, particularly in tubercular cases. It is the deprivation of them, together with insanitary surroundings and bad food, that renders the treatment of poor patients so tedious. When the necessity for recumbency has ceased, the patient should be sent away to Margate or one of the East Coast resorts, or failing those, to a somewhat elevated dry place in the country. But Margate air in tuberculosis is nearly a specific in itself. The food should be regular and plentiful, avoiding saccharine and starchy constituents in excess. The stomach and bowels must be regulated and constipation prevented. The best laxative in children is pulv. glycyrrhizæ ss. j at bedtime. Cod liver oil, cream, or one of the malt extracts are useful, and we may from time to time give iron in the form of syr. ferri phosphatis co., or syr. ferri. iodid ʒj thrice daily. In tubercular cases such general treatment is essential. In non-tubercular cases it assists their recovery most markedly.

*Treatment Direct to the Spine.*—The principles of treatment are three in number:—

(*a*) To fix the vertebral column, and to place it in the best possible circumstances for healing.

(*b*) To remove the weight of the upper part of the body from the diseased vetebræ.

(*c*) To prevent, as far as possible, unnecessary deformity by supporting the trunk, especially in front; and if deformity has occurred, to limit its increase.

To carry out these principles we have two methods at our disposal, viz: recumbency and the use of retentive appliances They may be employed separately or in combination in individual cases, but can never be used indiscriminately. The precise value of each varies according to the age, stage of the disease, and the different regions affected.

*Recumbency.*—Indications for:

1. In all acute cases in which there are considerable pain, distress and impairment of the general health.

2. When on employing the "palm-pressure" test to the back, it is found to be yielding anteriorly.

3. When paralysis and abscess are threatened.

4. Particularly in cases of severe cervical and lower lumbar caries.

5. In those patients who become easily tired on their feet, and in those who, apparently well supported mechanically, frequently desire to lie down.

6. In children recumbency may be resorted to with less danger to the general health than in adults. The immediate effects of recumbency are good. The pain disappears, the nervous irritability is lost, the face loses its anxious aspect, the patient often puts on fat, although the muscles of the limbs diminish in size.

The *advantages* are thus evident. The relief of pain, the limitation of the deformity and in some cases its recession, the gradual clearing up of any paresis, and the cessation of increase in the size of an abscess, are all good points.

The *disadvantages* are that in adults there is, after a comparatively short improvement, a marked decline in general health; anæmia and constipation ensue, and wasting again appears. In the case of children these are not marked. Provided the room be airy and sunshiny, young patients bear the prolonged lying down better than adults. But as caged animals are found to be liable to tubercular affections, so children deprived of their liberty will be unable to recover as rapidly as desirable from tubercular troubles. Formerly recumbency was persisted in for years, but now after a time we have the opportunity of giving patients more liberty by the use of plaster of Paris corsets, or some mechanical support such as a poroplastic jacket. It must be clearly borne in mind that when recumbency is demanded it must be absolute as long as it lasts.

The duration of recumbency can scarcely be specified in set terms of months. Each individual case must be judged on its merits. At the National Orthopedic Hospital I am accustomed to keep children recumbent until all pain has disappeared; until the palm-pressure best shows the back to be consolidated; and until, with a little support from the nurse, the child can be set up carefully for a few minutes, such raising to the vertical position not being accompanied nor followed by the pain. In cases which are doing well, the patient becomes restless, moves his arms and legs freely and fidgets constantly, or even attempts to turn over.

One finds, however, that unless the case is past treatment, the condition of the spine has markedly improved in three to nine months.

*Points to be noted in placing a patient in the recumbent position.*

1. The mattress should be of horsehair, firm, flat, and not too hard, pillows beneath the head being dispensed with.

2. Air-beds or water-beds are best avoided, on account of their instability. They are of doubtful service if the patient is very thin and bed-sores threaten. These may be obviated by alternating between the prone and supine positions.

3. It is necessary in most cases to employ some retentive arrangement to prevent movement. In cervical and dorsal cases a band across the chest, or a knitted-wool strap passing across the chest, with circlets for the arms, and fastening to the sides of the bed, is desirable. In diseases of the lumbar region it is necessary to supplement the upper band by a lower one across the abdomen. In older children the band acts as a moral rather than a physical restraint. In younger children it is really necessary. It is sometimes sufficient to pin the night-dress to the sheet, which is firmly fixed at the sides of the bed, or to use sandbags.

4. Should extension be used at the same time? If it be used with the idea of pulling apart the diseased vertebræ, the idea is erroneous, as a very large amount of force is necessary to separate the bodies; but if it be employed to relieve the pressure between the adjacent parts of the diseased and partially collapsed bodies, brought about by the action of irritated muscles, then its adoption is rational and necessary. In the cervical and upper dorsal forms of disease a leather collar and pulley may be fixed, and a weight of one to two pounds suspended, the head of the bed being raised one to two inches. The weight also serves to fix the head; or sand-bags may be used in addition if the case is an urgent one. In the mid and lower dorsal and lumbar regions, pads of soft felt may be placed beneath the body in the region of deformity, bearing on the soft tissues at the sides of the median line, and not on the spinous processes themselves; when the trouble is lower dorsal or lumbar, extension may be applied to the legs, the foot of the bed being elevated slightly. Such measures seem to me to answer most requirements, and I have not found it necessary to use Rauchfuss' suspensory cradle, although its merits are largely dwelt on by German and French surgeons.

5. In private cases it is desirable to have a couch or bed which can be moved and placed in a carriage, so that the patient may obtain fresh air. In poorer patients a Phelps' box answers the same purpose when the acuter symptoms have passed away.

6. The choice of positions—prone or supine? In the majority of cases the supine is preferable, although it may be replaced by the prone for a short time, to avoid bed-sores. Obviously in cervical disease where extension is necessary, the supine position alone is available. Redard* strongly advocates the prone position in those dorsal cases where the deformity is commencing, where there is a good deal of irritation and contraction of the muscles, and paralysis is setting in rapidly. He claims that in four out of eight cases he obtained a considerable diminution of the prominence, and in three that the deformity was not increased. He further states that the irksomeness of the position was soon overcome, and the general condition remained good, with no loss of appetite. The prone position has certainly the advantage in that it places the congested spine uppermost; but the disadvantages of it are that a specially constructed couch is necessary, and more continuous attention to the patient is required.

When the surgeon is satisfied that the severer symptoms have subsided, the spine is firm and the probability of abscess or other complications is slight, it is well to adopt a combination of the methods of partial recumbency and fixation appliances. This may be done by using either a double Thomas's hip splint and crutches, if the disease is low down in the spine or the patient can be firmly supported by one of the mechanical means to be detailed below. So that at first he is allowed to move about in the upright position for a few minutes daily, and the time is then gradually extended, more locomotion being allowed and less recumbency enforced; care being always taken that movement stops short of fatigue, and that during the time he is recumbent the horizontal position is strictly enforced. Various complicated arrangements are figured in books, and are in use, but I fail to see that they have any advantage either in the amount of movement allowed or support given over the simpler poroplastic or plaster jacket.

*Suspension.*—A few words are not out of place in dealing with this matter. It is absolutely essential in cervical disease, can be easily arranged by carrying the plaster bandages of a Sayre's jacket around the neck and forehead, leaving the face and vertex exposed. Naturally this is unsightly and cumbrous, but it affords excellent support. Or a jury-mast may be fitted to plaster or a poroplastic jacket. In the treatment of out-

* Traite de Chirurgie Orthoped pp. 244, 245.

patients I am accustomed to use a poroplastic jacket carrying a stem accurately adjusted to the dorsal curve and bearing an occipital head-rest, with a sling supporting the chin.

Another method is to supplement the felt jacket by a helmet and neck-piece, so making a complete cuirass after Walsham's plan. In lower cervical caries which are approaching cure it is often sufficient to continue the poroplastic material around the neck, and still later a simple felt or Thomas's leather collar may be used.

*Suggestions as to the Employment of Suspension.*—Suspension or support of the head and shoulders is necessary if the disease be above the fourth dorsal vertebra, and support of the shoulders alone if the disease be at or above the eighth dorsal. It is needful to insist upon this point, since one frequently sees cases of disease of the upper dorsal vertebræ brought to the hospital with nothing but a jacket on. The resulting increase of deformity is inevitable. In the dorsal region the natural backward curve is aggravated by the erosion of the bodies, and when the disease lies between the shoulder blades, they are separated from one another, the shoulders are displaced forward and the weight of the arms pulls the upper part of the vertebral column rapidly forward.

It appears to be a simple matter to speak so decidedly about, but its frequent neglect too often leads to serious consequences. An idea is too prevalent that for spinal disease anywhere except in the cervical region a Sayre's or poroplastic jacket is sufficient. Support, and in early cases backward traction of the arms are most essential to prevent unnecessary deformity of back and chest.

*Fixation and Supporting Appliances and their Principles and Tests of Efficiency.*—1. All complicated arrangements are to be avoided. In the plaster of Paris jacket properly applied, and to a considerable extent in the poroplastic jacket, all the needful requirements are fulfilled.

2. They must firmly fix the site of disease.

3. All weight must be taken off the affected region by transference of pressure from diseased to healthy spots.

4. They must be comfortable, cleanly and readily removable.

5. Pressure on the skin and chafing must be avoided.

6. They must be inexpensive and such as can be readily applied by the surgeon himself.

*Advantages of Plaster Jackets.*—1. When properly applied they are efficient in suitable cases.

2. The surgeon is independent of the instrument maker.

3. They cannot be removed at the whim of the patient.

4. Their cheapness.

*Disadvantages.*—1. They are apt to be uncleanly.

2. They become loose and badly fitting after a time.

3. They are apt to irritate the skin, especially over the prominences.

4. The condition of the back can not be watched.

5. If a discharging sinus is present it is difficult to keep it antiseptic.

*Poroplastic Jackets.*—These were introduced to supersede plaster of Paris.

*Advantages.*—1. Lightness and porosity.

2. Easy application in skilled hands.

3. Durability; they last for a year to eighteen months.

4. They permit greater cleanliness and allow the skin to be watched, and so chafing is prevented.

5. They set more rapidly than plaster and hence fixation is complete before release from suspension.

6. The plasticity, taking the shape of the figure exactly.

7. The same jacket can be remolded as often as necessary.

*Disadvantages.*—1. They are somewhat costly and out of the reach of poor patients.

2. They require considerable skill in adjustment, as they set so rapidly.

3. They are weak on the anterior aspect of the chest and over the crests of the ilia, i. e., two fixation points are not sufficiently firm.

*When May Treatment be Dispensed With in Spinal Caries?*—1. The absence of pain is no test, since pain naturally ceases if a support be worn, but if pain ensues on removal of the support the jacket must be put on again.

2. When the spine is firmly fixed and the deformity has remained stationary for some months.

3. If a recession of the deformity has been gained and maintained for some months.

4. If a compensatory lordosis just below the kyphosis is well established.

5. Dorsal caries is very rarely cured in one year; cervical and lumbar may require less.

6. If the improvement in the general health is sustained.

7. Supports must always be worn much longer in tubercular cases. If the support has been worn too long the muscles atrophy rapidly. In any case begin to dispense with the support gradually, especially if the patient is increasing in weight. (Ryan.)—*Pediatrics.*

